# Development and Evaluation of Short-Form Measures of the HIV/AIDS Knowledge Assessment Tool Among Sexual and Gender Minorities in Brazil: Cross-sectional Study

**DOI:** 10.2196/30676

**Published:** 2022-03-29

**Authors:** Rayanne C Ferreira, Thiago S Torres, Maria Das Graças B Ceccato, Daniel RB Bezerra, Brett D Thombs, Paula M Luz, Daphna Harel

**Affiliations:** 1 Escola Nacional de Saúde Pública Sérgio Arouca, Fundação Oswaldo Cruz Rio de Janeiro Brazil; 2 Instituto Nacional de Infectologia Evandro Chagas, Fundação Oswaldo Cruz Rio de Janeiro Brazil; 3 Universidade Federal de Minas Gerais Belo Horizonte Brazil; 4 Lady Davis Institute for Medical Research Jewish General Hospital Montreal, QC Canada; 5 Departments of Psychiatry McGill University Montreal, QC Canada; 6 Department of Epidemiology, Biostatistics and Occupational Health McGill University Montreal, QC Canada; 7 Department of Medicine McGill University Montreal, QC Canada; 8 Department of Psychology McGill University Montreal, QC Canada; 9 Biomedical Ethics Unit McGill University Montreal, QC Canada; 10 Department of Applied Statistics, Social Science, and Humanities New York University New York, NY United States; 11 Center for Practice and Research and the Intersection of Information, Society, and Methodology New York University New York, NY United States

**Keywords:** HIV, knowledge, sexual and gender minorities, Brazil, preexposure prophylaxis

## Abstract

**Background:**

In theoretical models of health behavior, knowledge about disease transmission and self-protective behaviors are conceptualized as important drivers of behavior change. Several studies conducted in Brazil point to an unfortunate convergence of sexual and gender minority (SGM) populations with low levels of HIV knowledge and younger age, lower education, engagement in higher-risk sexual behavior, and never having tested for HIV. Measures to assess level of HIV knowledge have been previously published, including the 12-item HIV/AIDS Knowledge Assessment (HIV-KA) tool. However, measure length can be a barrier to assessment.

**Objective:**

We started from the 12-item HIV-KA tool and developed candidate short forms using statistical procedures, evaluated their psychometric properties, and tested the equivalency of their associations with other measures of HIV knowledge compared to the 12-item version.

**Methods:**

A convenience sample of SGM was recruited during September 2020 to complete an online survey through advertisements on two social networking apps (Grindr and Hornet). The survey instrument included items on sociodemographic information, prior HIV testing and HIV test results, preexposure prophylaxis (PrEP) and antiretroviral treatment use, sexual behavior, and 3 HIV knowledge measures: the HIV-KA, World Health Organization Knowledge About HIV Transmission Prevention Indicator, and the Brief HIV Knowledge Questionnaire. We used exploratory factor analysis and confirmatory factor analysis (CFA) to assess the factor structure of the of the HIV-KA. We used optimal test assembly (OTA) methods to develop candidate short forms of the HIV-KA and evaluated them based on prespecified reliability, concurrent validity, and statistically equivalent convergent validity criteria.

**Results:**

Among 2552 SGM individuals from Brazil, mean age was 35.1 years, 98.2% (2507/2552) cisgender men and 1.8% (45/2552) transgender/nonbinary, 56.5% (1441/2552) White, and 31.0% (792/2552) self-reported HIV positive. CFA indicated a 1-factor structure for the 12-item HIV-KA. Concurrent validity correlations were high for all short forms with 6 items, but only versions with 9 items were as reliable as the full-length form and demonstrated equivalency for convergent validity correlations. Suggesting post hoc convergent validity, HIV knowledge scores using the 9- and 10-item short forms were higher for participants who perceived the Undetectable Equals Untransmittable (U=U) slogan as completely accurate versus not accurate. Suggesting post hoc concurrent validity, participants of younger age, of Black, Pardo or indigenous race, and reporting lower education and lower income scored lower on HIV knowledge. Participants who never tested for HIV scored lower than those who tested negative or positive, while those currently using PrEP scored higher than those reporting past or never use.

**Conclusions:**

OTA methods were used to shorten the 12-item HIV-KA to 9-item and 10-item versions while maintaining comparable reliability and validity among a large sample of Brazilian SGM. However, these short forms did not shorten sufficiently to justify deviation from the full measure.

## Introduction

Mirroring the dynamics of the HIV epidemic in Latin America, North America, and Western Europe, the Brazilian HIV epidemic is concentrated in key populations, such as gay, bisexual, and other men who have sex with men (MSM) [1,2] and transgender women [3]. In Brazil, the causes for this epidemiological profile are inherently multidimensional, involving vulnerability, risk, stigma, and discrimination, as well as behavioral, political, and programmatic issues [4,5]. Policies focusing on HIV prevention have incorporated HIV educational strategies as a main component as theoretical models of health behavior suggest that knowledge influences behavior [6]. More generally, knowledge about disease transmission and self-protective behaviors are conceptualized as important drivers of behavior change, thus influencing multiple decision-making models in the field of sexually transmitted infections, especially HIV [7].

Although mixed results exist on the direct link between knowledge and engagement in risky sexual behavior, a study conducted among men in Cape Town, South Africa, where HIV knowledge was low, showed that HIV testing combined with greater HIV knowledge led to reduced engagement in risky sexual behaviors [8]. Several studies conducted in Brazil point to an unfortunate convergence of sexual and gender minority (SGM) status with low level of HIV knowledge and younger age, lower education, engagement in higher-risk sexual behavior, and never having tested for HIV [9-12]. These findings highlight the vulnerabilities related to the social and cultural construction of the Brazilian HIV epidemic and the social inequalities. Moreover, these results indicate the important role that governmental and other stakeholder actions can have in improving indicators related to HIV knowledge.

Several measures to assess level of HIV knowledge have been published. A widely used measure is the World Health Organization Knowledge about HIV Transmission Prevention Indicator (WHO-KI), a 5-item measure proposed in a United Nations General Assembly Special Session that assesses knowledge of essential facts about transmission including correctly identifying ways of preventing sexual transmission of HIV and rejection of major misconceptions about HIV transmission [13]. Another measure is the Brief HIV Knowledge Questionnaire (HIV-KQ), a short version (18 items) of the 45-item HIV Knowledge Questionnaire, which has shown to be internally consistent, stable, and appropriate for individuals with low education. However, it may be considered somewhat outdated as it does not address new paradigms of HIV prevention and treatment, such as treatment as prevention [14] and pre- and postexposure prophylaxis [15]. In 2019, Guimarães et al [11] developed the HIV/AIDS Knowledge Assessment tool (HIV-KA), a 12-item measure in Brazilian Portuguese which includes items that address treatment as prevention and pre- and postexposure prophylaxis. The measure was applied in a large sample of gay, bisexual, and other MSM (n=4716) from 12 Brazilian cities, and results were analyzed using item response theory. Corroborating prior studies, individuals of higher socioeconomic status had a higher level of HIV knowledge.

The study of an individual’s sexual practices and its possible determinants, including HIV transmission knowledge, requires the concomitant measurement of multiple behaviors (eg, sexual behaviors, substance use) and knowledge-based and psychological constructs (eg, knowledge about transmission, perceptions of risk) which yield long study instruments. Importantly, we have observed that young, less-educated SGM who are most vulnerable to HIV acquisition are less likely to complete surveys [16]. Hence, shortening HIV knowledge instruments could help increase completion rate or allow for additional measures to be included in a given survey. Optimal test assembly (OTA) is a branch-and-bound, mixed-integer programming procedure that relies on estimates obtained from an item response theory model to select an optimal subset of items that best satisfy objective, reproducible, and prespecified constraints [17]. OTA was originally used in high-stakes large-scale educational assessments, but its use has been expanded to the creation of shortened forms of patient-reported outcomes [18-23]. This procedure has been shown to successfully produce replicable and reproducible shortened forms of minimal length [24].

This is a cross-sectional study of users of social networking apps for gay, bisexual, and other cisgender MSM and transgender and nonbinary or gender nonconforming individuals in Brazil. Our objective was to assess the psychometric properties of the HIV-KA among SGM and develop short forms of the tool using statistical procedures. To reach these objectives, we started with the 12-item HIV-KA from Guimarães et al [11] and developed candidate short forms, evaluated their psychometric properties, and tested the equivalency of their associations with other measures of HIV knowledge compared to the 12-item version. Using objective decision rules and two different convergent validity measures, we assessed two shortened forms, one per validity measure. Last, we used post hoc convergent validity to assess the properties of the two shortened forms.

## Methods

### Participants and Procedures

A convenience sample of SGM was recruited during September 2020 to complete an online survey through advertisements on two social networking apps (Grindr and Hornet). Grindr is a location-based social networking and online dating app launched in 2009 that has since become the largest and most popular gay mobile app in the world. Hornet, another location-based social networking and online dating platform, was launched in 2011 and is available as an app and on the web. For Grindr, advertisement banners were randomly displayed to users for 2 weeks. Hornet users received 1 inbox message with a link to the survey. Participants needed to provide electronic informed consent before initiating the survey. No compensation was provided, and no personally identifiable information was collected except for IP address. Participant eligibility included age 18 years and older and residency in Brazil. Exclusion criteria were self-identifying as a cisgender woman and an incorrect response to any of 3 attention questions that were included throughout the survey instrument at approximately every 15 items [25]. A full version of the survey instrument is provided in Multimedia Appendix 1.

### Ethics Approval

This study was approved by the National Institute of Infectious Diseases Programa Inova at the Oswaldo Cruz Foundation (FIOCRUZ) institutional review board (#CAAE 01777918.0.0000.5262) in accordance with all applicable regulations.

### Survey Instrument

The survey was programmed on Alchemer survey software. The survey was in Portuguese and contained 55 questions, with certain questions conditionally presented using branching logic. Survey links remained active for 1 month. Respondents were able to change and review answers. Four authors systematically checked the usability and technical functionality of the electronic questionnaire on different platforms and operating systems before starting the survey.

The survey instrument was divided into 3 sections. Section 1 included items on sociodemographic information (age, gender, sexual orientation, race/skin color, education, family monthly income, and state of residence). Section 2 included items referring to prior HIV testing and HIV test results. HIV-negative and unknown-status participants were questioned about preexposure prophylaxis (PrEP) use (current, never, or past), and those who were not currently using PrEP responded to 2 questions about sexual behavior during the previous 6 months (condomless anal sex and condomless receptive anal sex). HIV-positive participants were questioned about use of antiretroviral treatment (ART), and if in use, adherence (prior 7 days) was measured using the 3-item WebAd-Q instrument [26]. Section 3 included items of 3 HIV knowledge measures: HIV-KA [11], HIV-KQ [7], and WHO-KI [13]. These 3 measures use the same response format, which includes the options “true,” “false,” and “I don’t know.” The total score of each instrument was calculated by summing across all items that the participant answered correctly (“I don’t know” was coded as an incorrect response). Higher scores reflect greater knowledge. We also included a question about perceived accuracy of the Undetectable Equals Untransmittable (U=U) slogan [27-29]. Individuals were able to provide an email address if they wished to receive the correct answers to HIV knowledge questions 1 month after the study was terminated.

### Measures

HIV-KA [11] is a 12-item measure previously used to assess knowledge among 4176 MSM in 12 Brazilian cities with a respondent-driven sampling methodology used for recruitment. The tool was evaluated using item response theory with difficulty and discrimination parameters estimated by marginal maximum likelihood and the knowledge score (theta) estimated by the expected a posteriori method based on Bayesian statistical principles.

HIV-KQ [7] is an 18-item measure evaluated for its psychometric properties among low-income US adults (n= 1019). Results indicated strong levels of internal consistency and test-retest stability. For this study, we excluded 3 items: one was considered not relevant to the Brazilian context (“A natural skin condom works better against HIV than does a latex condom”), and 2 were deemed less relevant for SGM populations (“A woman cannot get HIV if she has sex during her period” and “There is a female condom that can help decrease a woman’s chance of getting HIV”).

WHO-KI [13] is a 5-item measure developed to assess progress in building knowledge of essential facts about HIV transmission among key populations. It is endorsed by the WHO and recommended as a tool to monitor key populations’ knowledge.

We assessed whether respondents correctly perceived the accuracy of the prevention benefits of U=U through the question “With regard to HIV-positive individuals transmitting HIV through sexual contact, how accurate do you believe the slogan Undetectable=Untransmissible is?” as used in previous studies [27-29]. Response options were based on a Likert-type scale from 1 (completely inaccurate) to 4 (completely accurate) plus a fifth option (I don’t know what “undetectable” means).

### Statistical Analysis

Descriptive statistics of the study population are provided. We randomly split the study population in half and assessed the factor structure of all items of the HIV-KA together as a single measure with exploratory factor analysis (EFA) in the first half followed by confirmatory factor analysis (CFA) in the second half. EFA was used to identify the number of factors and assess item factor loadings. EFA was performed using robust weighted least squares estimator given the categorical nature of the survey items, chi-square test statistic, and geomin oblique rotation [30]. A Cattell scree test on the sedimentation graph was examined. The number of factors was chosen based on the scree plot (eigenvalues), model adequacy, and overall interpretability. CFA used a weighted least squares estimator with a diagonal weight matrix, robust standard errors, and a mean- and variance-adjusted chi-square statistic with delta parameterization [31]. To assess model fit, the chi-square test, Tucker-Lewis Index (TLI) [32], comparative fit index (CFI) [33], root mean square error of approximation (RMSEA) [34], and standardized root mean residual (SRMR) [33] were used. Since the chi-square test is highly sensitive to sample size, it can lead to the rejection of well-fitting models [35]. Therefore, the TLI, CFI, and RMSEA fit indices were emphasized. Good fitting models may be indicated by a TLI and CFI ≥ 0.95, RMSEA ≤ 0.06, and SRMR < 0.08 [36].

Then, with the whole sample, a generalized partial credit item response theory model (GPCM) [37] was fit to all 12 items of the HIV-KA. The GPCM estimates 2 types of parameters for each item: a threshold parameter, which measures the level of knowledge at which people are more likely to answer the question correctly than incorrectly, and a discrimination parameter, which measures the strength of the association between that item and the underlying construct (HIV knowledge). From these item-level parameters, item information functions were estimated for each item and summed pointwise to obtain the test information function (TIF). The TIF measures the total amount of Fisher information in each item and is inversely related to the standard error of measurement of the underlying construct—that is, greater precision in the measurement of the underlying construct [38].

We used methods described by Harel et al [24]. Briefly, OTA systematically explores the space of all possible shortened versions of a fixed length to optimize the height of the TIF, thus minimizing the standard error of measurement of the underlying construct [24,39,40]. Here, for each possible length of shortened form (3 to 11), the OTA procedure created a candidate shortened version of the HIV-KA. Forms of length 1 and 2 were not generated because a minimum of 3 items are needed for the single-factor model to be identifiable [41]. Based on previously established guidelines [24], the OTA procedure was anchored at 5 points across the spectrum of the underlying construct (–5, –3, –1, 1, 3), jointly maximizing the shortened form’s TIF at these points [17].

Each of the candidate short forms and the full-length form were scored using two procedures to obtain estimates of each participant’s level of HIV knowledge. First, the summed scores across all items included in the form were calculated by adding item scores for each item included in the form. Second, factor scores, which estimate a level of a latent construct, were estimated from the GPCM for each participant for each form through an application of Bayes theorem. Both summed scores and factor scores were used due to the reliance on the former in research and the improved measurement properties of the latter [42,43].

Resulting from the OTA procedure are candidate shortened forms, each with the applicable optimal items. Removal of items implies a reduced amount of test information as compared to the full-length form. The selection of the final form was based on 5 criteria: reliability, concurrent validity based on summed scores, concurrent validity based on factor scores, convergent validity based on summed scores, and convergent validity based on factor scores. Applying these 5 criteria concurrently ensured that the final selected shortened version maintained desirable measurement properties across these categories.

Accordingly, we first generated the candidate shortened forms and assessed each form’s reliability against the full-length form using a Cronbach alpha coefficient [44]. The shortened version was required to maintain at least 95% of the value of Cronbach alpha for the full-length form. Second, we estimated concurrent validity for both summed and factor scores by calculating a Pearson correlation coefficient between the scores on each candidate shortened version and the scores on the full-length form. For both the summed and factor scores, these correlations were required to be at least 0.90, ensuring that the shortened version demonstrated high concurrent validity.

Next, for convergent validity, we used two different criteria based on 2 HIV knowledge measures (HIV-KQ and WHO-KI, Multimedia Appendix 2, Figure S1), and, in so doing, created 2 potential shortened forms—one validated against the HIV-KQ and the other validated against the WHO-KI. We assessed convergent validity through the correlation between participant scores on short forms and the 2 HIV knowledge measures. The candidate short forms were required to demonstrate statistical equivalence with the convergent validity of the full-length of each measure through an application of equivalence testing. Equivalence testing assesses whether the difference between 2 correlations is within a prespecified range, in this case set at .05 [45]. To assess statistical significance, we applied the Benjamini-Hochberg correction procedure for each of the hypothesis tests used (candidate shortened versions × 2 scoring procedures) [46].

Finally, post hoc convergent and concurrent validity of the shortened forms were evaluated. We calculated the mean scores and 95% confidence intervals when using the 2 candidate shortened forms according to the participant’s response to the perceived accuracy of the U=U slogan (dichotomized into completely accurate vs partially accurate, inaccurate, completely inaccurate, or “I don’t know what undetectable means”). Additionally, we calculated mean scores with 95% confidence intervals of the candidate shortened forms according to select variables. As observed in a prior study [11], we hypothesized that the scores of the shortened forms would be associated with age, race, education, and income. We also hypothesized that participants reporting access to HIV prevention services, measured by prior HIV testing [47] and PrEP use, would score higher in the shortened forms of the HIV-KA.

All analyses were conducted in R software (version 4.0.2, R Foundation for Statistical Computing) [48]. The GPCM was fit using the ltm package [49], and the OTA analysis was conducted using the lpSolveApi package [50].

## Results

### Descriptive Statistics of the Study Population

Of 3368 participants who initiated the survey, 2552 answered all items of the 3 HIV knowledge measures of interest and were included in analyses (Multimedia Appendix 2, Figure S2 and Table S1). Mean age of study participants was 35.1 years, with the overwhelming majority being cisgender men self-identified as gay or bisexual. Most participants self-reported as White, had a college education or higher, and were earning more than US $400 per month. Most (1510/2552, 59.2%) reported prior HIV testing and a negative HIV status; 31.0% (792/2552) self-reported as HIV positive. The vast majority (785/792, 99.1%) of HIV-positive participants had initiated ART, and, among these, most (472/785, 60.1%) were ART-adherent. Most (1534/1760, 87.2%) of HIV-negative or unknown participants were not currently using PrEP and reported condomless anal sex in prior 6 months (Table 1).

**Table 1:** Descriptive characteristics of participants included in the cross-sectional study among sexual and gender minorities, September 2020, Brazil.

			Value
Age (years), mean (SD)			35.1 (9.8)
**Age (years), median (IQR)**			33 (28-41)
	18-24	322 (12.6)	
	25-34	1069 (41.9)	
	35-44	722 (28.3)	
	45-54	311 (12.2)	
	55+	125 (5.0)	
**Gender, n (%)**			
	Cisgender man	2507 (98.2)	
	Transgender/nonbinary	45 (1.8)	
**Sexual orientation, n (%)**			
	Gay	2196 (86.1)	
	Bisexual	302 (11.8)	
	Hetero/pansexual/other	54 (2.1)	
**Race/skin color**			
	White	1441 (56.5)	
	Pardo	745 (29.2)	
	Black	297 (11.6)	
	Asian	28 (1.1)	
	Indigenous	18 (0.7)	
	Not declared	23 (0.9)	
**Education, n (%)**			
	Middle school	100 (3.9)	
	High school	671 (26.5)	
	College+	1765 (69.6)	
**Family monthly income (US $), n (%)**			
	Low (≤ 2 × minimum wage or ≤ 400)	752 (29.5)	
	Middle (> 2-6 × minimum wage or 401-1200)	1138 (44.6)	
	High (> 6 × minimum wage or > 1200)	662 (25.9)	
**Region, n (%)**			
	South/Southeast	2121 (83.1)	
	North/Northeast/Central-West	431 (16.9)	
**Recruitment, n (%)**			
	Grindr	1753 (68.7)	
	Hornet	737 (28.9)	
	Other	62 (2.4)	
**HIV test, n (%)**			
	Never tested	250 (9.8)	
	Negative	1510 (59.2)	
	Positive	792 (31.0)	
**ART^a^ self-report adherence (n=785, initiated ART), n (%)**			
	Yes	472 (60.1)	
	No	313 (39.9)	
**PrEP^b^ use (n=1760, HIV negative or never tested), n (%)**			
	Never	1412 (80.2)	
	Current	226 (12.8)	
	Past	122 (6.9)	
**Condomless anal sex (n=1760, HIV negative or never tested), n (%)**			
	Yes	717 (40.7)	
	No	1043 (59.3)	
**Measures of HIV knowledge, mean (SD)**			
	HIV-KA^c^	10.99 (1.46)	
	HIV-KQ^d^	13.07 (1.85)	
	WHO-KI^e^	4.76 (0.58)	
Perceived U=U^f^ slogan as completely accurate, n (%)			1600 (62.7)

^a^ART: antiretroviral therapy.

^b^PrEP: preexposure prophylaxis.

^c^HIV-KA: HIV/AIDS Knowledge Assessment tool.

^d^HIV-KQ: Brief HIV Knowledge Questionnaire.

^e^WHO-KI: World Health Organization Knowledge about HIV Prevention Indicator.

^f^U=U: Undetectable=Untransmittable.

### Factor Structure

The EFA of the 12-item HIV-KA (EFA sample; n=1276) yielded 1 eigenvalue of factor greater than 1 (Factor 1 eigenvalue 2.04). Based on examination of the scree plot, we judged that a 1-factor solution provided the most interpretable model. Results from the CFA (CFA sample; n=1276) indicated that a 1-factor structure showed reasonably good fit: χ^2^_66_=1352.1, *P*<.001; CFI=0.94; TLI=0.93; RMSEA=0.03; SRMR=0.11. The item loadings ranged from 0.18 (item 8) to 0.75 (item 5).

### Item Response Theory Model and OTA

The GPCM was fit on the 12 items of the HIV-KA. Item content with the discrimination parameters estimated from the GPCM are provided in Table S2 of Multimedia Appendix 2. The 3 items with the highest amount of discriminative ability and, therefore, the most influential on the TIF were items 2, 4, and 5. The items with the least amount of discriminative ability and, therefore, the least influential on the TIF were items 8, 9, and 11. Individual item information functions generated from the estimates of the GPCM and the test information function for the full-length form and the 2 short forms are provided in Figures S3 and S4, respectively, of Multimedia Appendix 2.

### Selection of the Final Shortened Version

Short forms with at least 8 items had Cronbach α=.59 or higher, suggesting a moderate level of reliability compared with full-length (for which Cronbach α=.64, Table 2). Short forms with the applicable items resulting from the OTA procedure are shown in Table S3 of Multimedia Appendix 2. Concurrent validity correlations were high for all short forms with 6 items or more based on factor score correlations (Table 2). For convergent validity, all versions with at least 9 items demonstrated statistically significant equivalency for the correlations between the summed and factor scores with the HIV-KA (Table 3). The 9-item shortened form was the shortest candidate version to fulfill our requirements when validating against the HIV-KQ for the equivalency analysis, while the 10-items shortened form was the shortest candidate when validating against the WHO-KI.

The selected short forms include, for example, the items addressing PrEP (item 1: “There are medications for HIV-negative people to take before having sex with other people to prevent HIV infection”), treatment as prevention (item 2: “An HIV-infected person who is taking HIV/AIDS medications has a lower risk of transmitting the virus to another person”), and postexposure prophylaxis (item 4: “There are medications for HIV/AIDS to be used after a situation of risk of infection [ie, unprotected sex, sexual violence]”; Table 3). The 3 items that were consistently dropped from the selected short versions were item 8 (“When having intercourse with only one faithful partner, not infected with HIV, the risk of contracting the virus is lower”), item 9 (“There is a cure for HIV”), and item 10 (“A healthy-looking person may be infected with the HIV virus”).

**Table 2:** Psychometric properties of the short forms of the HIV/AIDS Knowledge Assessment tool in the cross-sectional study among sexual and gender minorities, September 2020, Brazil.

Short form	Cronbach alpha	Correlation of summed scores with full form scores (95% CI)	Correlation of factor scores with full form score (95% CI)
3 items	.25	0.740 (0.722-0.758)	0.704 (0.684-0.723)
4 items	.42	0.798 (0.784-0.812)	0.803 (0.788-0.816)
5 items	.52	0.856 (0.846-0.866)	0.883 (0.874-0.891)
6 items	.56	0.889 (0.881-0.897)	0.918 (0.912-0.924)
7 items	.56	0.897 (0.889-0.904)	0.922 (0.916-0.928)
8 items	.59	0.919 (0.912-0.924)	0.941 (0.936-0.945)
9 items	.62	0.949 (0.945-0.953)	0.971 (0.968-0.973)
10 items	.63	0.962 (0.959-0.965)	0.980 (0.978-0.981)
11 items	.65	0.980 (0.978-0.981)	0.995 (0.995-0.996)
12 items	.64	1 (1-1)	1 (1-1)

**Table 3:** Convergent validity and equivalency analysis results for the HIV/AIDS Knowledge Assessment tool in comparison to the Brief HIV Knowledge Questionnaire and the World Health Organization Knowledge about HIV Prevention Indicator in the cross-sectional study among sexual and gender minorities, September 2020, Brazil.

					Equivalency analysis corrected *P* values		
Short form		Summed scores	Factor scores	Summed scores		Factor scores	
**Correlations with HIV-KQ^a^**							
	3 items	0.390 (0.357-0.423)	0.425 (0.392-0.456)	>.99		>.99	
	4 items	0.431 (0.399-0.462)	0.404 (0.371-0.436)	>.99		>.99	
	5 items	0.456 (0.425-0.486)	0.420 (0.388-0.451)	>.99		>.99	
	6 items	0.507 (0.478-0.536)	0.470 (0.439-0.500)	>.99		.83	
	7 items	0.511 (0.481-0.539)	0.474 (0.444-0.504)	.93		.53	
	8 items	0.526 (0.498-0.554)	0.496 (0.466-0.525)	.02		<.001	
	9 items	0.525 (0.496-0.553)	0.486 (0.455-0.515)	.01		<.001	
	10 items	0.536 (0.508-0.564)	0.495 (0.465-0.524)	<.001		<.001	
	11 items	0.566 (0.539-0.592)	0.520 (0.491-0.548)	<.001		<.001	
	12 items	0.561 (0.534-0.587)	0.521 (0.492-0.548)	<.001		<.001	
**Correlations with WHO-KI^b^**							
	3 items	0.396 (0.363-0.428)	0.418 (0.385-0.449)	>.99		>.99	
	4 items	0.430 (0.398-0.461)	0.395 (0.362-0.428)	>.99		>.99	
	5 items	0.416 (0.383-0.447)	0.369 (0.335-0.402)	>.99		>.99	
	6 items	0.457 (0.426-0.487)	0.415 (0.383-0.447)	>.99		>.99	
	7 items	0.472 (0.441-0.502)	0.426 (0.393-0.457)	>.99		>.99	
	8 items	0.566 (0.539-0.591)	0.508 (0.478-0.536)	<.001		<.001	
	9 items	0.538 (0.510-0.565)	0.476 (0.445-0.505)	.46		<.001	
	10 items	0.549 (0.521-0.575)	0.483 (0.453-0.512)	.002		<.001	
	11 items	0.561 (0.533-0.587)	0.496 (0.466-0.525)	.001		<.001	
	12 items	0.584 (0.558-0.609)	0.509 (0.480-0.537)	.001		<.001	

^a^HIV-KQ: Brief HIV Knowledge Questionnaire.

^b^WHO-KI: World Health Organization Knowledge about HIV Prevention Indicator.

### Post Hoc Convergent and Construct Validity of the Shortened Forms

HIV knowledge scores using both the 9-item and 10-item short forms were higher among participants who perceived the U=U slogan as completely accurate versus not accurate (Table 4). Participants of younger age, of Black, Pardo, or indigenous race, and reporting lower education and lower income scored lower on HIV knowledge. Participants who never tested for HIV scored lower than those who tested negative or positive, while those currently using PrEP scored higher than those reporting past or never use. Last, knowledge scores were very similar for those reporting condomless receptive anal sex or not as well as among those reporting as ART-adherent or not.

**Table 4:** Post hoc convergent and construct validities of the short forms of the HIV/AIDS Knowledge Assessment tool scales according to study variables in the cross-sectional study among sexual and gender minorities, September 2020, Brazil.

				9-item mean (95% CI)		10-item mean (95% CI)
**Convergent validity**						
	**Perceived U=U^a^ slogan as completely accurate**					
		Yes	8.49 (8.44-8.54)		9.46 (9.41-9.51)	
		No	7.86 (7.77-7.96)		8.83 (8.73-8.92)	
**Construct validity**						
	**Age (years)**					
		18-24	7.80 (7.63-7.97)		8.73 (8.54-8.91)	
		25-34	8.30 (8.23-8.37)		9.26 (9.19-9.34)	
		35-44	8.30 (8.22-8.39)		9.28 (9.20-9.37)	
		45-54	8.20 (8.07-8.33)		9.17 (9.04-9.31)	
		55+	7.94 (7.72-8.16)		8.92 (8.70-9.15)	
	**Race/skin color**					
		White or Asian	8.30 (8.24-8.35)		9.27 (9.21-9.33)	
		Black, Pardo, or indigenous	8.08 (8.00-8.16)		9.03 (8.95-9.12)	
	**Education**					
		Middle school	7.16 (6.76-7.56)		8.02 (7.59-8.45)	
		High school	7.89 (7.79-7.99)		8.85 (8.74-8.95)	
		College+	8.40 (8.35-8.45)		9.38 (9.33-9.43)	
	**Family monthly income**					
		Low	7.85 (7.74-7.96)		8.79 (8.68-8.91)	
		Middle	8.31 (8.25-8.38)		9.28 (9.22-9.35)	
		High	8.43 (8.36-8.50)		9.41 (9.34-9.48)	
	**HIV test**					
		Never tested	7.29 (7.07-7.52)		8.20 (7.96-8.44)	
		Negative	8.19 (8.13-8.25)		9.16 (9.10-9.23)	
		Positive	8.53 (8.46-8.59)		9.49 (9.43-9.56)	
	**PrEP^b^ use**					
		Never	7.98 (7.91-8.06)		8.94 (8.87-9.02)	
		Current	8.49 (8.38-8.60)		9.47 (9.36-9.58)	
		Past	8.20 (7.95-8.46)		9.16 (8.89-9.44)	
	**Condomless receptive anal sex**					
		Yes	8.09 (7.99-8.19)		9.05 (8.95-9.16)	
		No	8.05 (7.96-8.13)		9.01 (8.93-9.09)	
	**ART^c^ adherence**					
		Yes	8.58 (8.50-8.65)		9.54 (9.46-9.62)	
		No	8.47 (8.36-8.57)		9.44 (9.34-9.55)	

^a^U=U: Undetectable=Untransmittable.

^b^PrEP: preexposure prophylaxis.

^c^ART: antiretroviral therapy.

## Discussion

### Principal Findings

In this study, we used a novel OTA method to generate valid short-form measures of the HIV-KA. The OTA procedure generated 9-item and 10-item shortened forms that satisfied our prespecified criteria in terms of reliability, concurrent validity, and convergent validity. These versions maintained high reliability and high concurrent validity with the full-length form, as well as statistically equivalent convergent validity correlations with HIV-KQ and WHO-KI. Our results indicate that 9-item and 10-item HIV-KA versions could be used among Brazilian SGM to assess HIV knowledge. Nonetheless, unless the number of items is a critical issue for a particular study, we argue that the shortened forms were not short enough (only shortened by 2 to 3 items) to justify recommendation as these would not be directly comparable to existing studies that have used the full-length version.

The OTA method is a replicable method that maintains performance standards based on objective criteria and, as such, selected short forms can be said to fulfill prespecified reliability and concurrent and convergent validity. The reliability of the shortened forms was of the same magnitude as the full-length form while showing high concurrent validity with the full-length form. Statistically equivalent convergent validity correlations using 2 HIV knowledge measures (HIV-KQ and WHO-KI) were also shown for both summed and factor scores.

The selected short forms included the items addressing recent paradigms of HIV prevention and treatment, such as preexposure prophylaxis, treatment as prevention, and postexposure prophylaxis. The 3 items removed from the short forms showed the lowest discriminative ability, indicating that they were less useful in the construction of the HIV knowledge score. Notwithstanding, semantic aspects of the items may have impacted the results. Particularly, for item 8, the item with the lowest discriminative ability, the wording may have caused confusion. Item 8 states that the risk of infection is lower when it would have been more appropriate to say that the risk is null (“When having intercourse with only one faithful partner, not infected with HIV, the risk of contracting the virus is lower”). Moreover, the use of the word lower suggests that it is lower than some other situation that the item does not specify. For example, compared to complete sexual abstinence, the risk may be higher, although it is lower compared to other sexual behaviors.

Our results also indicate post hoc convergent validity for the shortened forms using the 1-item measure on the perceived accuracy of U=U, a slogan launched in 2016 by the Prevention Access Campaign to translate scientific evidence into a community message that highlights how people living with HIV on antiretroviral treatment with suppressed viral load cannot transmit HIV to their sexual partners [51]. The scientific evidence supporting U=U has accumulated over the past decade and is contingent on the body of knowledge showing effectiveness of treatment as prevention, in which the use of ART among people living with HIV reduces HIV transmission yielding public health as well as personal health benefits [14]. The observed convergent validity thus reinforces the value of the proposed shortened forms to measure HIV knowledge among SGM. Of note, mean HIV knowledge score was high considering all 3 measures, and the proportion of individuals perceiving U=U as accurate was higher than observed in a 2019 survey conducted among Brazilian gay, bisexual, and other MSM [29]. This may indicate that perceived accuracy of the U=U slogan actually increased among SGM from Brazil over time, an important positive finding as understanding the accuracy of U=U empowers those living with HIV, improving treatment adherence, and decreasing HIV-related stigma [52]. Additionally, it may also enhance scale-up of PrEP, which is available at no cost through the Brazilian public health system. However, there are significant sociodemographic differences between the sample populations, with the current sample having more participants from the South/Southeast of Brazil and with higher education and income. As such, these differences may also play a role explaining the increased perceived accuracy of U=U.

Although only slightly shorter than the original 12-item HIV-KA measure, the 9-item or 10-item shortened forms may be preferred as they reduce participant burden, which is particularly important for participants who may have difficulty completing self-reported questionnaires. As observed in this study (Multimedia Appendix 2, Table S1) and in previous online surveys conducted by our group among SGM, those aged 18 to 24 years reporting lower income and lower education who had never been tested for HIV were more likely to not complete the survey [16]. Future studies should assess whether using the 9-item or 10-item shortened forms ultimately reach SGM populations that are more sociodemographically diverse. The number of HIV cases among young gay, bisexual, and other MSM continues to rise in Brazil [53] and, although scarce, age-dependent HIV incidence rate estimates also show that younger gay, bisexual, and other MSM are the most vulnerable [54]. In this regard, the unbiased representation of gay, bisexual, and other MSM on surveys addressing HIV transmission knowledge and sexual behavior is paramount to improve and aid development of prevention campaigns to these groups. Furthermore, beyond objective knowledge, we echo the recent call for promoting prevention literacy, whereby knowledge of the multiple prevention modalities is promoted to allow individuals to make the decisions that are optimal for their health while also promoting community advocacy and mobilization [55]. Shorter questionnaires with accessible and appealing language constructed with community participation may increase completion rate, and, consequently, the value of the collected information particularly as applicable to vulnerable groups.

Ad hoc construct validity of the HIV-KA shortened forms showed that those of younger age, non-White race, reporting lower education and lower income scored lower on HIV knowledge. A study from Brazil on 4129 MSM recruited by respondent-driven sampling in 12 Brazilian cities in 2016 observed that not only was the prevalence of unprotected receptive anal intercourse higher among younger participants, they scored lower on HIV knowledge and were less likely to have been tested for HIV in the past, despite having more years of schooling [47]. This lower HIV knowledge may be a contributing factor to higher vulnerability to HIV infection. Indeed, multiple studies using different measures of HIV-related knowledge have shown a link between testing and knowledge with a gradient of increased knowledge as you move from the categories of never tested to HIV-negative and HIV-positive [28,29,56]. One hypothesis for this finding is that exposure to the health care setting and counseling during testing may increase HIV-specific knowledge. This rationale could also possibly explain why PrEP users who routinely have to access health services to refill their prescriptions also scored higher. That said, it is impossible to determine temporality and it may well be that those who are more knowledgeable about HIV are also more likely to get tested or use PrEP. We observed no correlation between HIV knowledge scores and report of condomless receptive anal sex, a finding also reported previously [56]. Future studies could explore motivations for engagement in high-risk sexual behavior and perhaps how HIV knowledge could help inform safer sexual practices.

### Limitations

There are several study limitations that must be considered. This study used cross-sectional data, and therefore the sensitivity to change and test-retest reliability of the HIV-KA short forms could not be assessed. All collected data were self-reported by participants and may be subject to measurement errors that can arise in the collection, recall, or recording of information. Participants were recruited from a convenience online sample and may not reflect other SGM populations in Brazil. As for any study design, online samples have strengths that should be acknowledged which include the reaching geographically diverse populations as well as individuals from remote regions and those completely disconnected from HIV prevention services [57]. The challenges include the need for testing of survey instrument on a variety of hardware devices and software platforms, for effective means of advertising to diverse populations and to maintain participant anonymity, among others [57]. Our participants were mostly cisgender, and additional studies should include a greater representation of transgender and nonbinary individuals. Additionally, the survey was advertised as about HIV knowledge and this may have influenced participant selection, possibly overrepresenting those already living with HIV. The OTA procedure is sensitive to the investigator-defined choice of decision criteria in the selection of the final shortened version. These decision criteria, when applied in future studies, must be carefully considered by researchers. Furthermore, the OTA method treats the 12 items of the HIV-KA as if they represented a full item bank of possible items. It is possible that if other items were considered than a different set of items would have been selected into the final short form. Lastly, this analysis should be replicated in other samples of SGM populations, as well as other populations, to increase the generalizability and to confirm that the selected short forms are optimal for other populations.

### Conclusion

In conclusion, this study showed how OTA methods might be used to shorten the 12-item HIV-KA to 9-item and 10-item versions while maintaining comparable reliability and validity among a large sample of Brazilian SGM. While OTA was primarily used for the development of high-stakes educational testing, it has now been used, as well, to successfully shorten patient-reported outcome measures in several patient populations. This study is the first, to our knowledge, to use OTA to shorten a knowledge assessment tool. Although the shortened forms in this study did not represent substantial reductions in items, OTA represents an important methodology as we attempt to maximize the information we collect in the least burdensome way possible, which can be supported by reducing the number of items in surveys.

## Appendix

Multimedia Appendix 1 Questionnaire.

Multimedia Appendix 2 Supplemental materials.

## Abbreviations

ART: antiretroviral treatment
CAPES: Coordenação de Aperfeiçoamento de Pessoal de Nível Superior
CFA: confirmatory factor analysis
CFI: comparative fit index
EFA: exploratory factor analysis
FIOCRUZ: Programa Inova at the Oswaldo Cruz Foundation
GPCM: generalized partial credit item response theory model
HIV-KA: HIV/AIDS Knowledge Assessment tool
HIV-KQ: Brief HIV Knowledge Questionnaire
MSM: men who have sex with men
OTA: optimal test assembly
PrEP: preexposure prophylaxis
RMSEA: root mean square error of approximation
SGM: sexual and gender minority
SRMR: standardized root mean residual
TIF: test information function
TLI: Tucker-Lewis Index
U=U: Undetectable=Untransmittable
WHO-KI: World Health Organization Knowledge About HIV Transmission Prevention Indicator
