# Supplementary material for: Development and Evaluation of Short-Form Measures of the HIV/AIDS Knowledge Assessment Tool Among Sexual and Gender Minorities in Brazil: Cross-sectional Study
Source: JMIR Public Health Surveill. 2022 Mar 29;8(3):e30676. doi: 10.2196/30676 (PMC9132367; doi:10.2196/30676)
Supplement: Multimedia Appendix 1 [file publichealth_v8i3e30676_app1.docx]

#### Multimedia Appendix 1. Questionnaire

#### 1) **What is your gender identity?**

( ) Cisgender man

( ) Transgender man

( ) Cisgender woman

( ) Transgender woman

( ) Non binary

( ) Travesti

( ) Other gender

#### 2) **What is your sexual orientation?**

( ) Asexual

( ) Bisexual

( ) Gay or Homosexual

( ) Heterosexual

( ) Lesbian

( ) Pansexual

( ) Other. Describe: _______________________________________________

### 3) How old are you (years)**?** ___________________________________

#### 4) **How did you find out about this project?**

( ) Hornet

( ) WhatsApp

( ) Other. Describe: _______________________________________________

#### 5) Do you currently have a steady partner?

( ) No

( ) Yes, and s/he is HIV negative

( ) Yes, and s/he is HIV positive

( ) Yes, and I don’t know if s/he is HIV negative or positive

#### 6) **In which State do you live?**

( ) Acre

( ) Alagoas

( ) Amapá

( ) Amazonas

( ) Sergipe

( ) Bahia

( ) Distrito Federal

( ) Ceará

( ) Espírito Santo

( ) Goiás

( ) Maranhão

( ) Mato Grosso

( ) Mato Grosso do Sul

( ) Minas Gerais

( ) Pará

( ) Paraíba

( ) Paraná

( ) Pernambuco

( ) Piauí

( ) Rio de Janeiro

( ) Rio Grande do Norte

( ) Rio Grande do Sul

( ) Rondônia

( ) Roraima

( ) Santa Catarina

( ) São Paulo

( ) Tocantins

( ) I don’t live in Brazil

#### 7) **Do you live in the capital city or in its metropolitan area?**

( ) Yes

( ) No

#### 8) **What is your level of education?**

( ) Incomplete elementary

( ) Complete elementary

( ) Complete Middle school

( ) Complete Superior

( ) Complete Graduation

( ) I don’t know or don’t want to answer

#### 9) What is your race or skin color?

( ) Asian

( ) White

( ) Indigenous

( ) *Pardo*

( ) Black

( ) I don’t know or don’t want to answer

#### 10) **What is your family monthly income?**

( ) No income

( ) up to 1039 reais (up to 1 minimum wage)

( ) 1040 to 2078 reais (>1 to 2 minimum wage)

( ) 2079 to 3117 reais (>2 to 3 minimum wage)

( ) 3118 to 4156 reais (>3 to 4 minimum wage)

( ) 4157 to 6234 reais (>4 to 6 minimum wage)

( ) 6235 to 10390 reais (>6 to 10 minimum wage)

( ) more than 10390 reais (>10 minimum wage)

#### 11) **Have you ever tested for HIV (lifetime)?**

( ) No, never. ***🡪 skip to question 17***

( ) Yes, but I don’t know the result. ***🡪 skip to question 17***

( ) Yes, and the last result was negative. ***🡪 skip to question 17***

( ) Yes, and the last result was positive

#### 12) Have you ever initiated anti-HIV treatment**?**

( ) Yes

( ) No ***🡪 skip to question 21***

#### 13) During the last 7 days, did you take your HIV drugs out of the scheduled indicated by your physician?

( ) Yes

( ) No

( ) I don’t know or don’t remember

#### 14) During the last 7 days, did you forget to take any of your HIV drugs**?**

( ) Yes

( ) No

( ) I don’t know or don’t remember

#### 15) During the last 7 days, did you take less or more pills of your HIV drugs**?**

( ) Yes

( ) No

( ) I don’t know or don’t remember

#### 16) Please indicate in the line below the point that express how well you took your HIV drugs during the last 30 days**.**

#### **For example:** **0% means you took no dose.** **50% means you took half of your doses.** **100% means you took all your doses.**

0 ________________________[__]_____________________________ 100

***🡪 skip to question 21 after answering question 16***

#### 17) Have your ever taken **PrEP (pre-exposure prophylaxys)?**

( ) I have never taken PrEP. ***🡪 skip to question 19***

( ) I am currently taking PrEP.

( ) I took PrEP in the past, but I am not taking it nowadays. ***🡪 skip to question 19***

#### 18) How are taking PrEP?

( ) I take one pill everyday.

( ) I take two pills at least 2 hours before sex, one pill 24h after and one pill 48h after (event-driven PrEP or 2:1:1)

( ) Other. Describe: _______________________________________________

#### 19) During the last 6 months, did you have condomless anal sex**?**

( ) Yes

( ) No ***🡪 skip to question 21***

#### 20) During the last 6 months, did you have receptive condomless anal sex**?**

( ) Yes

( ) No

#### 21) This question is just for verification. Please choose Option **C.**

( ) Option A ( ) Option B ( ) Option C ( ) Option D ( ) Option E

#### The next sentences address knowledge about HIV/AIDS. For each item, please answer whether true or false or if you don't know how to answer.

#### 22) **Coughing and sneezing do not spread HIV.**

( ) True ( ) False ( ) I don’t know

#### 23) **A person can get HIV by sharing a glass of water with someone who has HIV.**

( ) True ( ) False ( ) I don’t know

#### 24) **Pulling out the penis before a man climaxes/cums keeps a person from getting HIV during sex.**

( ) True ( ) False ( ) I don’t know

#### 25) **A person can get HIV if s/he has anal sex.**

( ) True ( ) False ( ) I don’t know

#### 26) **Showering or washing one's genitals/private parts (penis, vagina, and anus) after sex keeps a person from getting HIV.**

( ) True ( ) False ( ) I don’t know

#### 27) **All pregnant women infected with HIV will have babies born with AIDS.**

( ) True ( ) False ( ) I don’t know

#### 28) **People who have been infected with HIV quickly show serious signs of being infected.**

( ) True ( ) False ( ) I don’t know

#### 29) **There is a vaccine that can stop adults from getting HIV.**

( ) True ( ) False ( ) I don’t know

#### 30) **People are likely to get HIV by deep kissing, putting their tongue in their partner's mouth, if their partner has HIV.**

( ) True ( ) False ( ) I don’t know

#### 31) **A person will not get HIV if she/he is taking antibiotics.**

( ) True ( ) False ( ) I don’t know

#### 32) **Having sex with more than one partner can increase a person's chance of being infected with HIV.**

( ) True ( ) False ( ) I don’t know

#### 33) **Taking a test for HIV one week after having sex will tell a person is she/he has HIV.**

( ) True ( ) False ( ) I don’t know

#### 34) **A person can get HIV by sitting in a hot tub or a swimming pool with a person who has HIV.**

( ) True ( ) False ( ) I don’t know

#### 35) **A person can get HIV from oral sex.**

( ) True ( ) False ( ) I don’t know

#### 36) **Using vaseline or baby oil with condoms lowers the chance of getting HIV.**

( ) True ( ) False ( ) I don’t know

#### 37) This question is just for verification. Please choose Option **2.**

( ) Option 1 ( ) Option 2 ( ) Option 3 ( ) Option 4 ( ) Option 5

#### 38) **There are medications for HIV-negative people to take before having sex with other people to prevent HIV infection.**

( ) True ( ) False ( ) I don’t know

#### 39) **An HIV-infected person who is taking HIV/AIDS medications has a lower risk of transmitting the virus to another person.**

( ) True ( ) False ( ) I don’t know

#### 40) **An HIV-infected pregnant woman receiving HIV/AIDS medications during prenatal and at childbirth will have a lower chance of transmitting the virus to the baby.**

( ) True ( ) False ( ) I don’t know

#### 41) **There are medications for HIV/AIDS to be used after a situation of risk of infection (i.e. unprotected sex, sexual violence, etc).**

( ) True ( ) False ( ) I don’t know

#### 42) **People can be infected with HIV if they share utensils, cups, or meals.**

( ) True ( ) False ( ) I don’t know

#### 43) **People can be infected with HIV if they use public toilets.**

( ) True ( ) False ( ) I don’t know

#### 44) **People can be infected with HIV if it they are bitten by mosquitoes.**

( ) True ( ) False ( ) I don’t know

#### 45) **When having intercourse with only one faithful partner, not infected with HIV, the risk of contracting the virus is lower.**

( ) True ( ) False ( ) I don’t know

#### 46) **There is a cure for HIV.**

( ) True ( ) False ( ) I don’t know

#### 47) **A healthy-looking person may be infected with the HIV virus.**

( ) True ( ) False ( ) I don’t know

#### 48) **A person can contract HIV if he/she shares with other people instruments for the use of drugs such as syringes, needles etc.**

( ) True ( ) False ( ) I don’t know

#### 49) **People can contract HIV if they do not use condoms during sexual intercourse.**

( ) True ( ) False ( ) I don’t know

**Below we provide other questions about HIV/AIDS knowledge. Some sentences are similar, but it is important that you answer all of them. For each item, please answer whether true or false or if you don't know the answer.**

#### 50) **Can the risk of HIV transmission be reduced by having sex with only one uninfected partner who has no other partners?**

( ) True ( ) False ( ) I don’t know

#### 51) **Can a person reduce the risk of getting HIV by using a condom every time they have sex?**

#### ( ) True ( ) False ( ) I don’t know

#### 52) **Can a healthy-looking person have HIV?**

#### ( ) True ( ) False ( ) I don’t know

#### 53) **Can a person get HIV from mosquito bites?**

( ) True ( ) False ( ) I don’t know

#### 54) **Can a person get HIV by sharing food with someone who is infected?**

( ) True ( ) False ( ) I don’t know

#### 55) **With regard to HIV+ individuals transmitting HIV through sexual contact, how accurate do you believe the slogan "Undetectable = Untransmissible" is?**

( ) Completely accurate ( ) Somewhat accurate ( ) Somewhat inaccurate ( ) Completely inaccurate ( ) I don’t know what undetectable means

## Thanks!
