# Supplementary material for: Development and Evaluation of Short-Form Measures of the HIV/AIDS Knowledge Assessment Tool Among Sexual and Gender Minorities in Brazil: Cross-sectional Study
Source: JMIR Public Health Surveill. 2022 Mar 29;8(3):e30676. doi: 10.2196/30676 (PMC9132367; doi:10.2196/30676)

**Multimedia Appendix 2.** Supplemental materials.

**Development and evaluation of short-form measures of the HIV/AIDS Knowledge Assessment tool among sexual and gender minorities in Brazil: a cross-sectional study**

**Authors**

**Table of contents:**

Table S1

Table S2

Table S3

Figure S1

Figure S2

Figure S3

Figure S4

**Table S1.** Descriptive characteristics of the participants reached with the survey stratified by those who partially completed and completed the survey instrument in the cross-sectional study among sexual and gender minorities, September 2020, Brazil.

|  | Partially completed | Completed |
| --- | --- | --- |
|  | **N=502** | **N=2552** |
| Age (years) |  |  |
| 18-24 | 102 (20.3) | 322 (12.6) |
| ≥ 25 | 400 (79.7) | 2230 (87.4) |
|  |  |  |
| Gender |  |  |
| Cisgender men | 490 (97.6) | 2507 (98.2) |
| Transgender/non-binary | 12 (2.4) | 45 (1.8) |
|  |  |  |
| Sexual orientation |  |  |
| Hetero/pansexual/other | 22 (4.4) | 54 (2.1) |
| Gay | 393 (78.6) | 2196 (86.1) |
| Bisexual | 85 (17) | 302 (11.8) |
|  |  |  |
| Race/skin color |  |  |
| Asian | 1 (0.2) | 28 (1.1) |
| White | 203 (49.8) | 1441 (56.5) |
| Indigenous | 6 (1.5) | 18 (0.7) |
| *Pardo* | 134 (32.8) | 745 (29.2) |
| Black | 59 (14.5) | 297 (11.6) |
| Not declared | 5 (1.2) | 23 (0.9) |
|  |  |  |
| Education |  |  |
| Middle-school | 59 (14.7) | 100 (3.9) |
| High-school | 119 (29.7) | 671 (26.5) |
| College+ | 223 (55.6) | 1765 (69.6) |
|  |  |  |
| Family monthly income |  |  |
| Low (≤ 2 minimum wages or ≤ USD 400) | 198 (48.6) | 752 (29.5) |
| Middle (> 2-6 minimum wages or USD 401-1200) | 149 (36.6) | 1138 (44.6) |
| High (> 6 minimum wages or > USD 1200) | 60 (14.7) | 662 (25.9) |
|  |  |  |
| Region |  |  |
| North/Northeast/Central-west | 81 (19.8) | 431 (16.9) |
| South/Southeast | 328 (80.2) | 2121 (83.1) |
|  |  |  |
| HIV test |  |  |
| Never tested | 72 (18.8) | 250 (9.8) |
| Negative | 199 (51.8) | 1510 (59.2) |
| Positive | 113 (29.4) | 792 (31) |
|  |  |  |
| Recruitment |  |  |
| Grindr | 280 (68.5) | 1753 (68.7) |
| Other | 18 (4.4) | 62 (2.4) |
| Hornet | 111 (27.1) | 737 (28.9) |

**Table S2.** Item content and discrimination parameters of the HIV/AIDS Knowledge Assessment tool (HIV-KA) in the cross-sectional study among sexual and gender minorities, September 2020, Brazil.

| Item content | N (%) correct | Discrimination |
| --- | --- | --- |
| 1. There are medications for HIV-negative people to take before having sex with other people to prevent HIV infection. | 2117 (83.0) | 1.215 |
| 2. An HIV-infected person who is taking HIV/AIDS medications has a lower risk of transmitting the virus to another person. | 2158 (84.6) | 1.761 |
| 3. An HIV-infected pregnant woman receiving HIV/AIDS medications during prenatal and at childbirth will have a lower chance of transmitting the virus to the baby. | 2071 (81.2) | 1.566 |
| 4. There are medications for HIV/AIDS to be used after a situation of risk of infection (i.e. unprotected sex, sexual violence, etc). | 2388 (93.6) | 2.015 |
| 5. People can be infected with HIV if they share utensils, cups, or meals. | 2445 (95.8) | 2.053 |
| 6. People can be infected with HIV if they use public toilets. | 2390 (93.7) | 1.537 |
| 7. People can be infected with HIV if it they are bitten by mosquitoes. | 2347 (92.0) | 1.309 |
| 8. When having intercourse with only one faithful partner, not infected with HIV, the risk of contracting the virus is lower. | 2305 (90.3) | 0.523 |
| 9. There is a cure for HIV. | 2335 (91.5) | 1.155 |
| 10. A healthy-looking person may be infected with the HIV virus. | 2508 (98.3) | 1.689 |
| 11. A person can contract HIV if he/she shares with other people instruments for the use of drugs such as syringes, needles etc. | 2462 (96.5) | 1.216 |
| 12. People can contract HIV if they do not use condoms during sexual intercourse. | 2521 (98.8) | 1.433 |

**Table S3.** Items included in each of the 9 short-forms of the HIV/AIDS Knowledge Assessment tool (HIV-KA) in the cross-sectional study among sexual and gender minorities, September 2020, Brazil.

| Short form | 1 | 2 | 3 | 4 | 5 | 6 | 7 | 8 | 9 | 10 | 11 | 12 |
| --- | --- | --- | --- | --- | --- | --- | --- | --- | --- | --- | --- | --- |
| 3-items |  | Y |  |  | Y |  |  |  |  | Y |  |  |
| 4-items |  | Y |  | Y | Y |  |  |  |  | Y |  |  |
| 5-items |  | Y | Y | Y | Y |  |  |  |  | Y |  |  |
| 6-items |  | Y | Y | Y | Y | Y |  |  |  | Y |  |  |
| 7-items |  | Y | Y | Y | Y | Y |  |  |  | Y |  | Y |
| 8-items |  | Y | Y | Y | Y | Y | Y |  |  | Y |  | Y |
| 9-items* | Y | Y | Y | Y | Y | Y | Y |  |  | Y |  | Y |
| 10-items** | Y | Y | Y | Y | Y | Y | Y |  |  | Y | Y | Y |
| 11-items | Y | Y | Y | Y | Y | Y | Y |  | Y | Y | Y | Y |
| 12-items | Y | Y | Y | Y | Y | Y | Y | Y | Y | Y | Y | Y |

Y = included in the short-form

*Short form chosen when using HIV-KQ

**Short form chosen when using WHO-KI

Figure S1. Schematic representation of study procedures showing how the HIV/AIDS Knowledge Assessment tool (HIV-KA) was evaluated and how, for convergent validity, two different criteria were used based on two HIV knowledge measures (the Brief HIV Knowledge Questionnaire [HIV-KQ] and the WHO knowledge about HIV prevention indicator [WHO=KI]) in the cross-sectional study among sexual and gender minorities, September 2020, Brazil.


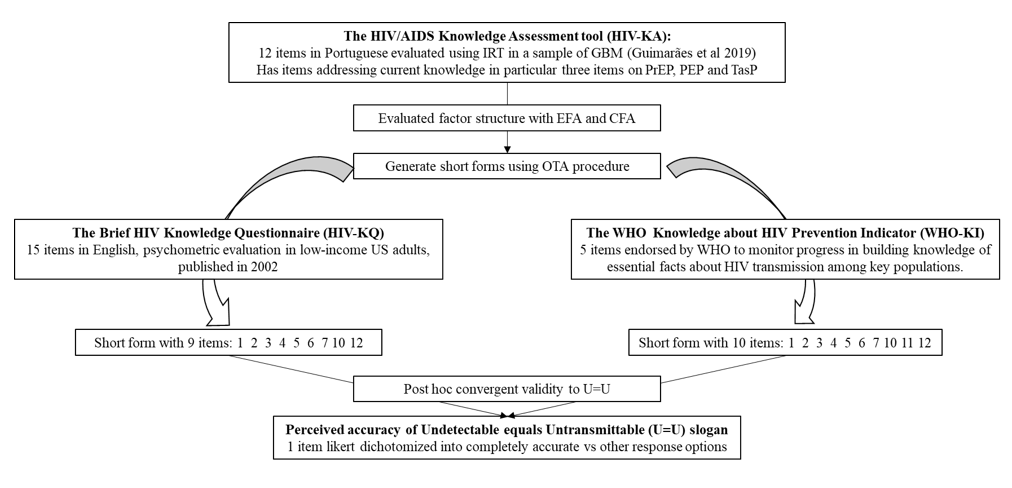


Figure S2. Flow chart of study participants included in the cross-sectional study among sexual and gender minorities, September 2020, Brazil.


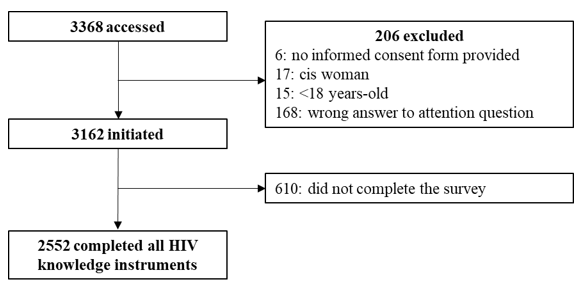


Figure S3. Individual item information functions generated from the estimates from the generalized partial credit item response theory model (GPCM) in the cross-sectional study among sexual and gender minorities, September 2020, Brazil.


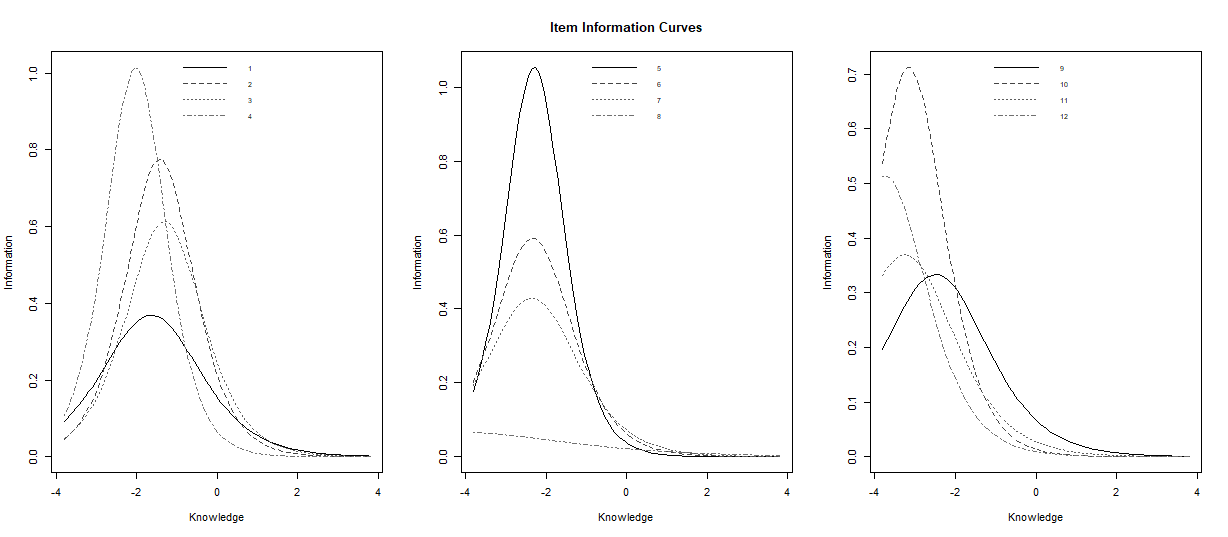


Figure S4. Test information function for the full-length and selected short-forms in the cross-sectional study among sexual and gender minorities, September 2020, Brazil.


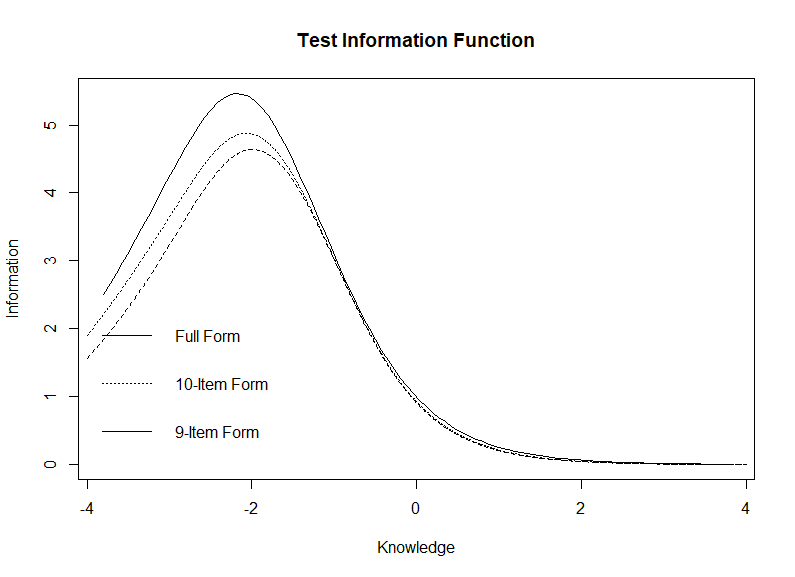

Supplement: Multimedia Appendix 2 [file publichealth_v8i3e30676_app2.docx]
